# Adiponectin Signaling Regulates Lipid Production in Human Sebocytes

**DOI:** 10.1371/journal.pone.0169824

**Published:** 2017-01-12

**Authors:** Yu Ra Jung, Jin-Hyup Lee, Kyung-Cheol Sohn, Young Lee, Young-Joon Seo, Chang-Deok Kim, Jeung-Hoon Lee, Seung-Phil Hong, Seong-Jun Seo, Seong-Jin Kim, Myung Im

**Affiliations:** 1 Department of Dermatology, College of Medicine, Chungnam National University, Daejeon, Korea; 2 Department of Dermatology, College of Medicine, Dankook University, Cheonan, Korea; 3 Department of Dermatology, Chung-Ang University College of Medicine, Seoul, Korea; 4 Department of Dermatology, Chonnam National University Medical School, Gwangju, Korea; San Gallicano Dermatologic Institute, ITALY

## Abstract

Adiponectin plays important roles in metabolic function, inflammation and multiple biological activities in various tissues. However, evidence for adiponectin signaling in sebaceous glands is lacking, and its role remains to be clarified. This study investigated the role of adiponectin in lipid production in sebaceous glands in an experimental study of human sebocytes. We demonstrated that human sebaceous glands *in vivo* and sebocytes *in vitro* express adiponectin receptor and that adiponectin increased cell proliferation. Moreover, based on a lipogenesis study using Oil Red O, Nile red staining and thin layer chromatography, adiponectin strongly upregulated lipid production in sebocytes. In three-dimensional culture of sebocytes, lipid synthesis was markedly enhanced in sebocytes treated with adiponectin. This study suggested that adiponectin plays a significant role in human sebaceous gland biology. Adiponectin signaling is a promising target in the clinical management of barrier disorders in which sebum production is decreased, such as in atopic dermatitis and aged skin.

## Introduction

Adiponectin, an adipokine expressed specifically in adipose tissue, has been implicated in various diseases, such as diabetes, obesity and cardiovascular disease. It functions primarily as a metabolic mediator of insulin sensitivity and glucose homeostasis, and plays a role in regulating inflammation in immune responses [1, 2]. In addition, recent reports revealed an important role of adiponectin in tissue regeneration, as adiponectin deficiency significantly inhibits the mechanisms underlying tissue renewal [3].

Adiponectin acts as an important signal in skin physiology and function. It enhances the proliferation and migration of keratinocytes and adiponectin-deficient mice display impaired re-epithelialization [4]. In addition, adiponectin is a hair growth stimulator that increases the secretion of growth factors from dermal papillae cells and hair matrix keratinocyte proliferation [5]. Moreover, adiponectin induces proliferation of dermal fibroblasts and upregulation of collagen production [6] and promotes hyaluronan synthesis with increased hyaluronan synthase [7, 8]. The age-dependent decreasing pattern of adiponectin in skin suggests the possibility that adiponectin plays a role in skin aging [8].

The sebaceous gland plays a vital role in skin homeostasis by producing sebum. Sebocytes lipids are secreted to the skin surface, where they function as an important component of the skin barrier with keratinocyte-derived lipids [9]. Defective lipid synthesis in sebocytes is a predisposing factor for the development of skin inflammatory diseases including atopic dermatitis and psoriasis [10]. Sebaceous glands express both adiponectin receptors (AdipoR)1 and AdipoR2 [5], and this finding suggested a role of adiponectin in sebum production. However, no reports have shown that adiponectin signaling directly influences lipid production. The potential roles of adiponectin and its mechanisms of action in human sebaceous glands require clarification. In this study, we investigated the effects of adiponectin on sebum production in the sebaceous glands *in vitro*. Our findings provide compelling evidence that adiponectin plays a significant role in human sebocyte biology.

## Materials and Methods

### Reagents

Recombinant human full-length adiponectin was obtained from Biobud (Seongnam, Korea). For Western blotting and immunohistochemical analysis, we used specific antibodies against the following proteins: AdipoR1, AdipoR2, Akt, sterol response element binding protein (SREBP)-1, actin (Santa Cruz Biotechnologies, Santa Cruz, CA, USA), APPL, phospho-AMP activated protein kinase (AMPK), AMPK, phospho-Akt (Cell Signaling, Danvers, MA, USA) and adiponectin (Abcam, Cambridge, UK). Palmitic acid, squalene and compound C were purchased from Sigma-Aldrich (St. Louis, MO, USA).

### Ethics statement

Specimens for sebocyte culture and immunohistochemistry were obtained from the faces of patients undergoing surgical procedures. Written informed consent was obtained, and all experiments were carried out in accordance with the ethical committee approval process of the Institutional Review Board of Chungnam National University Hospital (CNUH 2015-02-013).

### Cell culture

Primary cultures of sebocytes from 3 different donors were established according to a method described previously [1]. Briefly, human sebaceous glands were isolated from the separated epidermis under a microscope and transferred to tissue culture dishes. The cells were maintained in Sebomed medium (Biochrom, Berlin, Germany) supplemented with 10% fetal bovine serum (Gibco BRL, Rockville, MD, USA) and 5 ng/mL recombinant human epidermal growth factor (Invitrogen, Carlsbad, CA, USA) at 37°C in a humidified atmosphere containing 5% CO_2_. After the cells became subconfluent, they were harvested with 0.05% Trypsin-EDTA (Gibco BRL) and subcultured. Isolated primary sebocyte cultures in this study were characterized in terms of sebaceous functionality. The squalene and wax esters, which could hardly be found at epidermal keratinocytes, were extracted from primary sebocytes (S1A Fig) and the expression of human epithelial membrane antigen (EMA) and keratin (KRT) 7 were enhanced in primary sebocytes whereas KRT 5 and involucrin showed low expression compare to keratinocytes. Primary sebocytes were used at passages ranging from 3 to 6 in all experiments. The pre-confluent cells were treated with adiponectin at 3–4 days after passage and post-confluent cells at 7–10 days.

### Immunohistochemistry

Tissue samples were fixed with 10% formaldehyde, embedded in paraffin, and cut into sections at a thickness of 4 μm. The sections were deparaffinized in xylene and then rehydrated through an alcohol series. Antigens were retrieved by pressure-cooking the sections in citrate buffer (10 mM citric acid, pH 6.0) for 4 min. Endogenous peroxidase activity was blocked by incubating the sections with 2% hydrogen peroxide for 30 min at room temperature. The primary antibodies were AdipoR1 (goat polyclonal, Santa Cruz SC-46748, 1:100), AdipoR2 (goat polyclonal, Santa Cruz SC-46751, 1:100), and EMA (mouse monoclonal, Dako, 1:100), and then incubated with secondary antibody at room temperature for 30 min. The sections were incubated with diaminobenzidine tetrachloride solution at room temperature for 5 min and counterstained with Mayer’s hematoxylin.

### Immunocytofluorescence

Primary sebocytes were grown on coverslips, fixed with 4% paraformaldehyde for 20 min, and permeabilized with 0.1% Triton X-100 in PBS for 10 min at room temperature. Cells were incubated overnight at 4°C with primary antibody and then for 1 h at room temperature with FITC-conjugated secondary antibody. Primary antibodies were AdipoR1, AdipoR2 and SREBP-1 (rabbit polyclonal, Santa Cruz SC-367, 1:100). Secondary antibodies were donkey anti-goat IgG Alexa Fluor 488-labeled and goat anti-rabbit IgG Alexa Fluor 568-labeled (Invitrogen, 1:200). Finally, cells were visualized under a fluorescence microscope (Olympus Corp., Tokyo, Japan).

### Western blotting

Cells were lysed in Proprep solution (Intron, Daejeon, Korea). Samples were resolved by sodium dodecyl sulfate-polyacrylamide gel electrophoresis (SDS-PAGE), transferred onto nitrocellulose membranes, and incubated with appropriate antibodies. The following primary antibodies were AdipoR1, AdipoR2, APPL1 (rabbit monoclonal, Cell Signaling 3858, 1:1000), AMPKα (rabbit polyclonal, Cell Signaling 2532, 1:1000), phospho-AMPKα (rabbit polyclonal, Cell Signaling 2531, 1:1000), Akt (rabbit monoclonal, Cell Signaling 4691, 1:1000), phospho-Akt (rabbit polyclonal, Cell Signaling 9271, 1:1000), SREBP-1, adiponectin (mouse monoclonal, Abcam ab22554, 1:1000), and actin(goat polyclonal, Santa Cruz SC-1615, 1:1000) as a loading control. Blots were then incubated with peroxidase-conjugated secondary antibodies and visualized by enhanced chemiluminescence (Intron). Protein intensity was quantified using IMT i-Solution software (IMT Inc., Daejeon, Korea), and the values obtained were normalized relative to the actin signal.

### Reverse transcription polymerase chain reaction (RT-PCR)

To evaluate gene expression, total RNA was extracted using an easy-BLUE RNA extraction kit (Intron). Total RNA (2 μg) was reverse-transcribed using M-MLV reverse transcriptase (ELPIS Biotech, Daejeon, Korea). Aliquots of the reverse-transcribed mixture were subjected to PCR using specific primer sets as follows: AdipoR1, forward 5'-CTTCTACTGCTCCCCACAGC-3' and reverse 5'-ACAAAGCCCTCAGCGATAG-3'; AdipoR2, forward 5'-CTTCTACTGCTCCCCACAGC-3' and reverse 5'-ATGGCCAGCCTCTACATCAC-3'; and GAPDH (control), forward 5'-CGCTCTCTGCTCCTCCTGTT-3' and reverse 5'-CCATGGTGTCTGAGCGATGT-3'.

### Assessment of cell viability

Human sebocytes (2 × 10^5^ cells per well) were seeded onto six-well plates. The next day, the cells were treated with various doses of adiponectin and subsequently incubated for 24 h. The ratio of cytotoxicity was assessed using 5 mg/mL of MTT reagent (3-(4,5-dimethylthiazol-2-yl)-2,5-diphenyltetrazolium bromide) for 2 h, after which precipitated formazan was dissolved with dimethyl sulfoxide and the optical density (OD) was measured at 540 nm using a spectrophotometer.

### Cell growth analysis

[^3^H]-Thymidine uptake assay was performed to determine the effects of adiponectin on proliferation of human sebocytes. Sebocytes in 12-well culture plates were treated every 2 days with various doses of adiponectin for a total of 6 days. Cells were incubated with fresh medium containing 1 μCi of [^3^H]-thymidine (Amersham, Buckinghamshire, UK). Following incubation for the indicated times, cells were washed twice with PBS and incubated with 0.1 N NaOH at room temperature. Levels of radioactivity in cell lysates were measured using a liquid scintillation counter and recorded as a measure of cell proliferation. In addition, we performed low-density seeding assays. Briefly, cells were seeded at a density of 10^2^ cells per well in six-well plates and allowed to grow for 6 days. Micrographs were obtained on each day to determine relative cell growth rates.

### Lipid detection

To detect sebaceous lipids, primary sebocytes were seeded at a density of 3 × 10^4^ cells per well onto 12-well culture plates and incubated overnight, after which they were treated every 2 days with various doses of adiponectin for a total of 6 days. Cells were washed with PBS and fixed with 10% formaldehyde at room temperature for 60 min. Fixed cells were stained for 60 min with filtered Oil Red O (Sigma) working solution, prepared immediately before use by making a 6:4 mixture of stock (0.5% Oil Red O in 99% isopropanol) and distilled H_2_O. Stained cells were washed in distilled H_2_O, counterstained with hematoxylin, and visualized by microscopy. For quantitative detection of intracellular lipids, Oil Red O was removed by incubating the cells with 100% isopropanol, and supernatant Oil Red O levels were determined by measuring the OD at 500 nm. To calculate the amount of lipid per cell, the OD value was normalized relative to the cell count and determined using a FACSCanto^™^ II flow cytometer^®^ and FACSDiva software (BD Biosciences, San Jose, CA, USA). For Nile red staining, a stock solution of Nile red (Sigma-Aldrich; 1 mg/mL in acetone) was diluted to a final concentration of 1 μg/mL in PBS. Cells were fixed in 4% formaldehyde at room temperature for 5 min, stained with Nile red solution for 15 min at 37°C and washed with PBS. Stained cells were visualized by fluorescence microscopy using 485-nm excitation and 565-nm emission filters.

### Thin layer chromatography (TLC)

The composition of sebocytes in major neutral lipids was investigated by separating crude lipid extracts by TLC. A total of 2 μCi of [^14^C]-acetic acid (Perkin-Elmer Corp., Norwalk, CT, USA) was added to the medium for 6 h. After removing the medium, cells were detached and transferred into 1.5-mL tubes containing 200 μL of a chloroform/methanol (2:1) mixture. After cell debris was removed by centrifugation at 13,000 rpm for 5 min, the tubes were dried using a vacuum evaporator. The lipid components extracted from sebocytes were analyzed by chromatographic separation on 20-cm silica gel TLC plates that had been previously charged with chloroform. After spotting the lipids (40 μL) dissolved in chloroform, the plates were developed three times as follows: (1) hexane to the top, (2) toluene to the top, and (3) hexane/ether/acetic acid (70:30:1) 10 cm from the top. A 30-min drying period in a standard fume hood was performed between each mobile phase to ensure complete evaporation of the solvent.

### Three-dimensional (3D) culture

We performed 3D culture of human sebocytes using Matrigel (BD Technology, Durham, NC, USA) according to a method described previously [11]. Matrigel (50–100 μL) was added to glass-bottom dishes (diameter of 1 cm) and allowed to set during incubation for 20 min on ice. Primary sebocytes in 400 μL of medium and 2% matrigel were then added to the matrigel layer. The overlay medium was replaced every 2 days during culture of the cells for 7 days.

### RNA interference

Small-interfering RNAs (siRNAs) were chemically synthesized, annealed and transfected into 60–70% confluent sebocytes. A total of 20 nM AdipoR1, AdipoR2 siRNA and negative control siRNA were transfected into the sebocytes using Lipofectamine 2000 (Invitrogen) according to the manufacturer’s instructions. The sequences of the sense siRNAs are as follows: AdipoR1 GGACAACGACUAUCUGCUACATT; AdipoR2 GGAGUUUCGUUUCAUGAUCGGTT. After 24 h of transfection, the cells were treated with 200 ng/ml of human adiponectin and harvested.

### Adenovirus creation

An aliquot of reverse transcription (RT) mixture was subjected to PCR cycles with the primer set for adiponectin (forward 5'-TTGGGATCCATGCTGTTGCTGGGAGCTG-3' and reverse 5'-TGCCTCGAGTCAGTTGGTGTCATGGTAG-3'). The amplified full-length cDNA for adiponectin was subcloned into the pENT/CMVGFP vector that had attL sites for site-specific recombination with a Gateway destination vector (Invitrogen). Replication-incompetent adenoviruses were created using the Virapower adenovirus expression system (Invitrogen). Site-specific recombination between entry vector and adenoviral destination vector was achieved by LR clonase (Invitrogen). The resulting adenoviral expression vector was then transfected into 293A cells using Lipofectamine 2000 (Invitrogen). Cells were grown until 80% cytotoxic effect was seen, then harvested for preparation of recombinant adenovirus.

### Co-culture of keratinocytes with sebocytes

For culture of primary keratinocytes, human skin samples were treated with dispase overnight at 4°C. The epidermis was separated and placed in a solution containing 0.05% trypsin and 0.025% EDTA for 15 min at 37°C. After vigorous pipetting, cells were pelleted and resuspended in keratinocyte growth medium (KGM). In co-culture with sebocytes in cell culture inserts, primary sebocytes in inserts (5 × 10^5^ per 0.4 μm pore inserts [Nunc A/S, Roskilde, Denmark]) were incubated with primary keratinocytes (2 × 10^5^ per well in six-well culture plates) with DMEM/Ham’s F12 medium (Invitrogen). Likewise, primary keratinocytes incubated with sham inserts served as controls. To evaluate the epidermal barrier markers, keratinocytes were trypsinized and retrieved from wells after removing the sebocytes and control sham inserts.

### Real-time PCR

Aliquots of an RT mixture were analyzed by quantitative real-time RT–PCR using specific primer sets. The following primer sequences were used: involucrin, forward 5'-CCACTGGCTCCACTTATTTCG-3' and reverse 5'-GGACAGAGTCAAGTTCACAGA-3'; filaggrin, forward 5'-CGAAGGAGCCAAAAATATAAA-3' and reverse 5'-GATGTGCTAGCCCTGATGTTG-3'; fatty acid synthase, forward 5'-AGTACACACCCAAGGCCAAG-3' and reverse 5'-GGATACTTTCCCGTCGCATA-3'; HMG-CoA reductase, forward 5'-GTCATTCCAGCCAAGGTTGT-3' and reverse 5'- CTTTGCATGCTCCTTGAACA-3'; PPAR-γ, forward 5'-CGCCCAGGTTTGCTGAATGTG-3' and reverse 5'-ACTCATGTCTGTCTCCCGTCTTCTTTG-3';EMA, forward 5'-TGAGCCAGTACCCCACCTAC-3' and reverse 5'-ACCTGAGTGGAGTGGAATGG-3'; and fatty acid desaturase 2 (FADS2), forward 5'-ATCCCTTTCTACGGCATCCT-3' and reverse 5'-CCACTGAACCAGTCGTTGAA-3'. For quantitative real-time RT–PCR, SYBR Green real-time PCR master mix (Invitrogen) was used according to the manufacture’s protocol.

### Statistical analysis

All experiments were repeated at least three times with different batches of cells from different donors. Data were evaluated statistically using Student’s *t* test. In all analyses, *P* < 0.05 was taken to indicate statistical significance.

## Results

### Adiponectin receptor expression in human sebaceous glands and primary sebocytes

**Fig 1:**
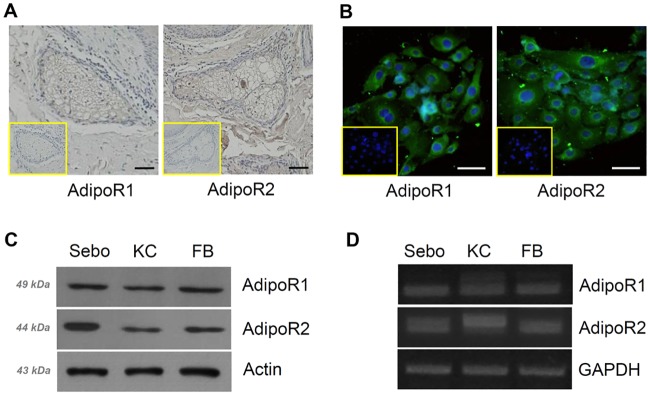
Expression of adiponectin receptors in human sebaceous glands and sebocytes. (A) Human sebaceous glands were assayed for adiponectin receptor (AdipoR)1 and AdipoR2 by immunohistochemistry. Inset, isotype control. (B) Immunofluorescence labeling of AdipoR1 and AdipoR2 (green) in human sebocytes. Nuclei were counterstained with DAPI (blue). Inset, isotype control. (C, D) Western blotting and RT-PCR of sebocyte lysates. Human keratinocytes and fibroblasts expressing AdipoR1 and AdipoR2 were used as positive controls. Scale bars = 20 μm. Sebo, sebocytes; KC, keratinocytes; FB, fibroblasts.

As a preliminary study, we examined the expression of AdipoR1 and AdipoR2 in human sebaceous glands. Immunohistochemical analysis revealed the presence of these receptors in normal human sebaceous glands (Fig 1A), which was consistent with a previous report [5]. We then assessed the presence of adiponectin receptors in human sebocytes. The results of immunocytochemical, Western blotting, and RT-PCR analyses indicated that human sebocytes express AdipoR1 and AdipoR2 at the protein and mRNA levels (Fig 1B–1D). As a positive control, the adiponectin receptor was probed and detected in normal human keratinocytes and fibroblasts, which have been reported to express this receptor [4, 6]. These results suggested a possible role of adiponectin in human sebocyte biology.

### Adiponectin induces proliferation of human sebocytes

**Fig 2:**
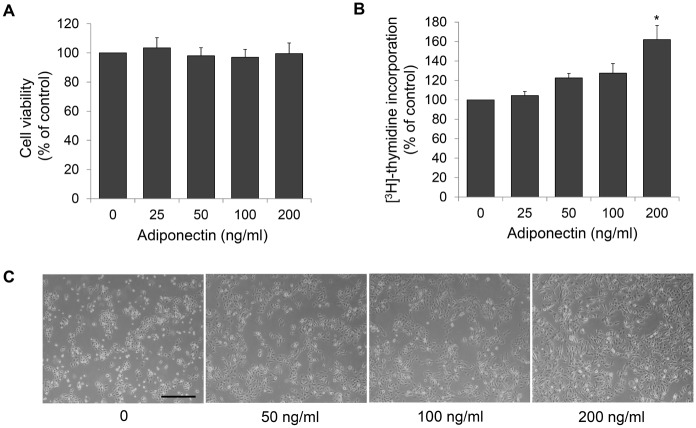
Effects of adiponectin on cell proliferation in human sebocytes. (A) The effects of adiponectin on sebocyte viability were examined by MTT assay. (B) Rate of [^3^H]-thymidine incorporation into sebocytes treated with various doses of adiponectin, calculated as a percentage of the value in untreated cells. (c) Dose-dependent effects of adiponectin on sebocyte proliferation were examined using microscopy from low cell seeding assays. Scale bars = 400 μm. Data represent means ± SEM (*n* = 6). Data were analyzed using Student’s *t* test (*P < 0.05).

Primary sebocytes were treated with adiponectin to explore the potential role of adiponectin *in vitro*. We first examined the cytotoxicity of adiponectin in sebocytes by MTT assay. Adiponectin at various doses up to 200 ng/mL exerted no cytotoxic effects on cultured sebocytes (Fig 2A). We then examined the effects of adiponectin on proliferation of sebocytes by [^3^H]-thymidine incorporation assay, and the results indicated significant induction of proliferation at 200 ng/mL adiponectin (Fig 2B). In the low cell seeding assays, adiponectin also increased the growth rate of human sebocytes at 200 ng/mL (Fig 2C). These observations suggested that adiponectin efficiently induces the proliferation of human sebocytes in *vitro*.

### Adiponectin enhances lipid synthesis in human sebocytes

**Fig 3:**
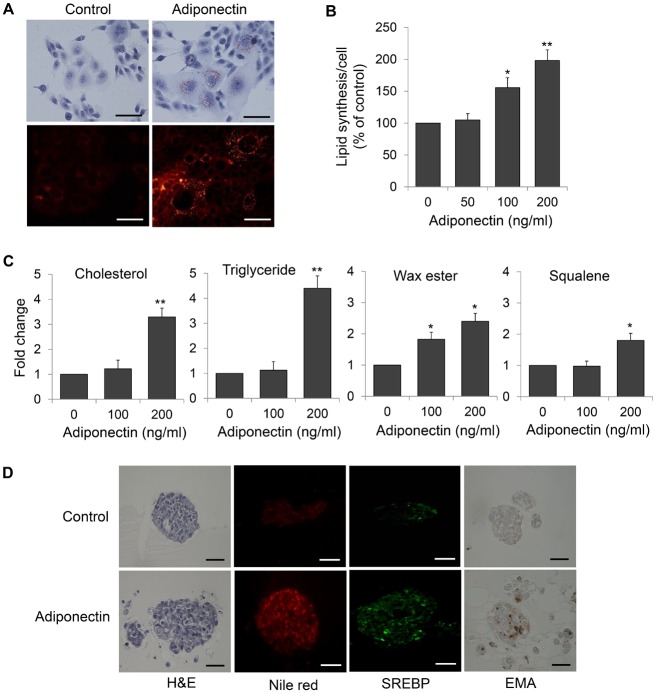
Effects of adiponectin on lipid production in human sebocytes. (A) Detection of intracellular lipids in sebocytes treated with adiponectin using microscopy after Oil Red O and Nile red staining. Scale bars = 20 μm. (B) Lipid levels in sebocytes treated with various doses of adiponectin, calculated as percentages of the value of untreated cells. (C) Relative abundance of major lipid classes determined by thin-layer chromatography. Human sebocytes grown in the presence of [^14^C]-acetate after treatment with adiponectin, and changes in specific lipid components such as cholesterol, triglyceride, wax ester, and squalene, were analyzed. (D) The effect of adiponectin in three-dimensional (3D) culture of human sebocytes. Sebocytes in 400 μL of medium were added to the matrigel layer (50–100 μL) adding to glass-bottom dishes. Sebocytes were treated with 200 ng/mL adiponectin and the overlay medium was replaced every 2 days during culture of the cells for 7 days. Organoid structures in 3D culture of sebocytes were stained with hematoxylin and eosin (H&E), Nile red, SREBP and epithelial membrane antigen (EMA). Scale bars = 20 μm. Data represent means ± SEM (*n* = 8). Data were analyzed using Student’s *t* test (*P < 0.05, **P < 0.01).

Previous studies indicated that confluent sebocytes contained more intracellular lipid droplets with differentiated sebocytes [12, 13, 14]. Therefore, we used pre-confluent undifferentiated sebocytes to explore the role of adiponectin when sebaceous gland activity decreased. When sebocytes were treated with 200 ng/mL adiponectin, increased lipid accumulation in the cytoplasm was detected by microscopy after staining with Oil Red O or Nile red (Fig 3A). In addition, adiponectin induced intracellular lipid levels in a dose-dependent manner as determined by measuring the OD (Fig 3B). Next, we used TLC analysis to further examine the relative abundance of major neutral lipid classes. Adiponectin significantly up-regulated all types of lipids including cholesterols, triglycerides, wax esters and squalene (Fig 3C). In order to confirm the role of adiponection in terms of sebaceous lipid droplet production, we have performed additional experiments with SZ95 sebocytes and confirmed that adiponectin enhances lipid synthesis in SZ95 sebocytes too (S2 Fig). To recapitulate the organization of sebaceous glands *in situ*, we developed a 3D culture system using matrigel. Human sebocytes maintained in 3D culture formed organoids that mimicked the organization of sebaceous glands *in situ*. Organoids from sebocytes exposed to adiponectin showed a larger and more complex structure and increased lipid expression by Nile red staining. In addition, SREBP1 and EMA, a potential marker of lipid production in human sebocytes, were also enhanced by adiponetin treatment (Fig 3D).

### Adiponectin increases lipid synthesis in human sebocytes via APPL1-AMPK signaling

**Fig 4:**
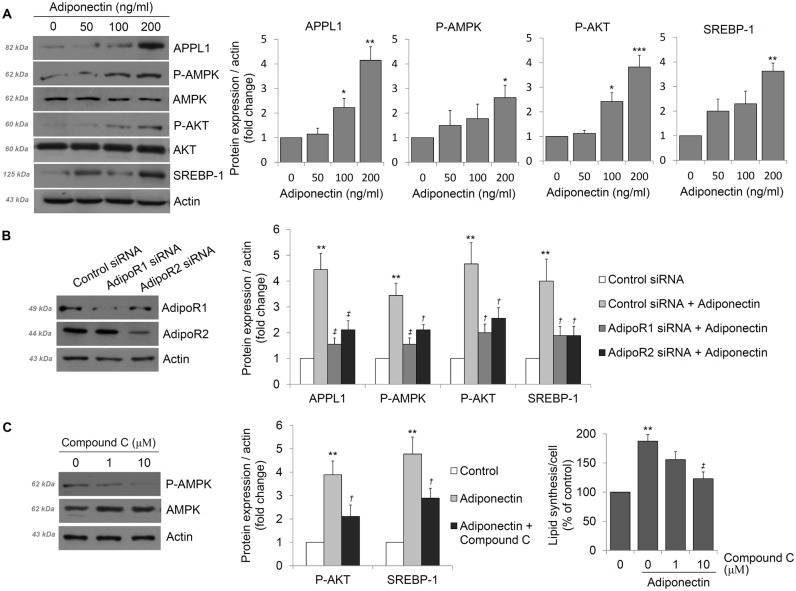
Induction of lipid synthesis by adiponectin in human sebocytes by activating APPL1-AMP activated protein kinase (AMPK) signaling. (A) After sebocytes were treated with adiponectin, whole cell lysates were prepared and analyzed by Western blotting. Blots were incubated with specific antibodies. Densitometric analyses of these protein signals were normalized relative to those for actin controls. (B) Sebocytes were transfected with control, AdipoR1 or AdipoR2-targeting siRNA and the intracellular protein levels of each signals were determined by Western blotting. (C) Sebocytes were incubated with different concentrations of compound C and protein signals were determined by Western blotting and intracellular lipid levels were measured by Oil Red O staining. Data represent means ± SEM (*n* = 6). Data were analyzed by Student’s *t* test (*P < 0.05, **P < 0.01, ***P < 0.001 *vs*. untreated cells; †P < 0.05, ‡P < 0.01 *vs*. adiponectin-treated cells).

Based on the above results, we evaluated the molecular mechanism by which adiponetin enhances lipid synthesis. A number of studies indicated that binding of adiponectin to AdipoR1 and AdipoR2 promotes APPL1-AMPK signaling in a variety of cell types and transmits a range of signals from the cell surface to intracellular targets [1, 15]. Levels of APPL1 in human sebocytes increased after adiponectin treatment, and phosphorylated AMPK expression was upregulated by adiponectin in a dose-dependent manner. We further examined Akt, which is an intracellular molecule influenced by AMPK signaling [15, 16], and found that the levels of phosphorylated Akt were significantly induced by adiponectin. In addition, levels of SREBP-1, which promotes the lipogenesis of human sebocytes by activation of Akt [17], increased after adiponectin treatment (Fig 4A). To confirm these molecular pathways, we generated a siRNA specific for AdipoR1 and AdipoR2 to knock-down expression of these genes. As shown in Fig 4B, AdipoR1 or AdipoR2 siRNA significantly reduced the protein level such as APPL1, phospho-AMPK, phospho-Akt and SREBP-1 which were increased by adiponectin treatment, whereas a scrambled control siRNA did not (Fig 4B). Moreover, compound C, a pyrrazolopyrimidine derivative that acts as a selective ATP-competitive inhibitor of AMPK [18], was found to reduce the protein level such as phospho-Akt and SREBP as well as lipid production after adiponectin stimulation. These results suggested that adiponectin increases APPL1-AMPK signaling and that this upregulation may be correlated with induction of lipogenesis in human undifferentiated sebocytes *via* the Akt/SREBP pathway.

## Discussion

Recent evidence has indicated that adiponectin has a causal role in the pathophysiology in several skin diseases. Adiponectin could negatively regulate psoriasis progression. Serum adiponectin levels are decreased in patients with psoriasis, and its levels are inversely correlated to the severity of disease and concomitant diseases such as metabolic syndrome [19, 20]. In addition, adiponectin regulates psoriasiform skin inflammation by suppressing IL-17 production from γδ-T cells [21]. Low adiponectin levels were associated with increased prevalence of atopic dermatitis and its aggravation was associated with changes in the plasma concentration of adiponectin [22, 23]. In addition, adiponectin is a potent mediator in the regulation of cutaneous wound healing. The administration of adiponectin accelerated wound healing via upregulation of keratin and ameliorated impaired wound healing in diabetic mice [4, 24]. A recent report showed that adiponectin is one of several downregulated genes in sensitive skin, and its reduced expression might be responsible for the induction of pain sensation in sensitive skin [25]. Although adiponectin affects skin functions under various conditions, little information is available on the effect of adiponectin on skin lipid synthesis. To the best of our knowledge, this is the first study to investigate the role of adiponectin in sebaceous gland-derived lipids.

Several previous reports support our findings. In an *in vivo* study, adiponectin promoted the formation of differentiated sebaceous gland structures with an increase in the number of sebaceous glands [4]. Another report demonstrated that high fat diets reduce lipid synthesis in the skin through lowered adiponectin activity [26], providing indirect evidence that adiponectin is associated with a positive relationship in skin lipid synthesis. In lipid metabolism of adipocytes, adiponectin leads to the accumulation of a greater number of larger lipid droplets in adipocytes. As an autocrine factor in adipose tissues, adiponectin promoted cell proliferation and differentiation from preadipocytes into adipocytes, augmenting the gene expression responsible for adipogenesis [27]. As shown in S3 Fig, overexpression of adiponectin using adenovirus demonstrated to induce APPL1, phospho-AMPK, phospho-Akt, and SREBP-1 in a manner similar to exogenously added adiponectin. This is based on the assumption of the autocrine secretion of adiponectine by sebocytes which has reported in recent paper [28]. Finally, these results support our data, which showed adiponectin-induced upregulation of lipogenesis in sebocytes.

The major function of lipids in skin is to form a permeability barrier between the hostile external environment and the internal milieu; therefore, alteration in the metabolism of these lipids can lead to skin dysfunction [10, 29]. Sebum lubricates the skin and traps moisture. Stratum corneum hydration declines in areas of human skin having decreased sebaceous glands, and glycerols derived from triglycerides in sebaceous glands play an important role in skin hydration [30, 31]. In addition, sebum protects against ultraviolet and is a source of antioxidants, anti-inflammatory and antimicrobial peptides [9, 32]. Our supplemental data suggested that sebocyte lipids could positively influence the skin barrier function. As shown in S1 Fig, keratinocytes co-cultured with sebocytes in inserts showed a significant increase in the expression of genes involved in the barrier formation of keratinocytes, such as involucrin, filaggrin, fatty acid synthase and HMG-CoA reductase comparing to only keratinocytes culture (S4A Fig). In addition, palmitic acid, the most abundant fatty acid in sebum, induced expression of these genes in a dose-dependent manner. Squalene, which is characteristically found in sebum and serves as a marker to distinguish sebaceous from keratinocyte lipids, displayed results similar to those obtained with palmitic acid (S4B Fig). Other lipids such as triglycerides, wax ester, oleic acid and linoleic acid were not influence to expression of these genes in keratinocytes. However, the FAS is responsible for the de novo synthesis of palmitate and HMG-CoA reductase is the enzyme involved in the initial steps of the squalene biosynthesis. Therefore, excess of enzymatic product would exert a feedback inhibition of the biosynthetic pathway. Further investigation regarding the role and fundamental mechanism of these lipids from sebaceous gland in the skin permeability barrier is required.

The dysfunction of lipid synthesis in sebaceous glands may be a contributor to the pathogenesis of inflammatory dermatoses. In atopic dermatitis, decreased sebum production is commonly associated with reduced skin hydration. Sebocyte proliferation and sebaceous gland activity are reduced significantly in atopic dermatitis [33, 34], suggesting a link between decreased sebaceous gland function and skin barrier dysfunction in atopic dermatitis. Scalp psoriatic lesions show significant atrophy in sebaceous glands, with decreased number and size. In addition, it is associated with thinner hair in patients with acute inflamed psoriatic scalp skin [35]. Aging skin displays morphological changes and alterations in sebaceous glands. The sebum secretion rate reaches its maximum throughout the teens, remains steady until middle age, and tends to decline slowly in old age [36, 37]. These physiologic changes showing reduced sebaceous gland activity correlate with the period in which the aging adult develops xerotic dermatitis. Collectively, these findings suggest that there are many possible medical applications of adiponectin for treating various barrier dysfunctions such as atopic dermatitis, scalp psoriasis and aging-related xerosis.

To investigate the promoting effect of adiponectin in conditions of decreased lipogenesis, we used undifferentiated sebocytes showing reduced sebaceous gland activity. However, excessive sebum production induces the formation of lesions associated with acne [38]. Therefore, we further examined whether adiponectin influences differentiated sebocytes with sebaceous glands under normal conditions. Early differentieated sebocytes showed large cells without detectable cytoplasmic droplets and mid differentiated sebocyets exhibited the cells with some cytoplasm and perinuclear distribution of cytoplasmic droplets. And late differentiated sebocytes demonstrated the abundant cytoplasm and perinuclear distribution of cytoplasmic droplets. Comparing to pre-confluent sebocytes, post-confluent sebocytes demonstrated the relative fully differentiated sebocytes showing more abundant lipid synthesis and up-regulated the expression of genes potentially related to sebocyte differentiation (S5A Fig). And then the adiponectin treatment did not increased the lipogenesis of differentiated sebocytes in Oil red O and TLC analysis (S5B Fig). In other words, adiponectin could be effective only when sebum production is insufficient, suggesting the possibility that adiponectin could help maintain equilibrium of a plateau concentration that would exert biological effects.

This study demonstrated that adiponectin receptors are expressed in human sebocytes and that adiponectin induced proliferation and lipid synthesis in human sebocytes and the 3D culture model. Our findings suggest that sebaceous glands are the direct target of adiponectin and that adiponectin may play an important role in lipid metabolism in the sebaceous gland. Moreover, adiponectin signaling is a promising target in the clinical management of barrier dysfunction showing hyposeborrhoeic states. Further clinical studies investigating the effect of adiponectin in skin disorders will help clarify the role of adiponectin in sebaceous glands.

## Supporting Information

S1 Fig Sebaceous functionality of isolated primary sebocytes. (A) Thin-layer chromatography of neutral lipids extracted from epidermal keratinocytes, primary sebocytes, SZ95 sebocytes, Lipids were identified by comparison with lipid standards. (B) Quantitative reverse transcription polymerase chain reaction analysis of various genes showing sebocytes characteristics. Data represent means ± SEM (*n* = 7). Data were analyzed by Student’s *t* test (*P < 0.05, **P < 0.01). KC, keratinocytes; Sebo, primary sebocytes; SZ95, SZ95 sebocytes; EMA, epithelial membrane antigen; KRT, keratin; NS, no significant difference.
(TIF)

S2 Fig Induction of lipid production by adiponectin in the SZ95 sebocytes. (A) Intracelluar lipid levels in SZ95 sebocytes treated with various doses of adiponectin using Red O staining, calculated as percentages of the value of untreated cells. (B) Relative abundance of lipid determined by thin-layer chromatography. SZ95 sebocytes grown in the presence of [14C]-acetate after treatment with 200ng/ml adiponectin, and changes in specific lipid components were analyzed. Data represent means ± SEM (*n* = 5). Data were analyzed by Student’s *t* test (*P < 0.05).
(TIF)

S3 Fig Increase of APPL1-AMPK signaling by overexpression of adiponectin. Sebocytes were transduced with an adenovirus expressing adiponectin (Ad/Adipo) or LacZ (control) and expression of related genes was also determined by Western blot analysis. Densitometric analyses of these protein signals were normalized relative to those for actin controls. Data represent means ± SEM (*n* = 5). Data were analyzed by Student’s *t* test (*P < 0.05, **P < 0.01).
(TIF)

S4 Fig Sebocyte lipids induced the expression of genes related to the epidermal barrier. (A) To evaluate the effect of sebocytes lipids on epidermal barrier, keratinocytes from the same donor with sebocytes incubated with and without sebocytes in inserts Quantitative reverse transcription polymerase chain reaction analysis of various genes expressed in keratinocyte and sebocyte co-cultures. (B) Changes in gene expression in keratinocytes after treatment with palmitic acid and squalene, which are major components of sebum in human sebaceous glands. Data represent means ± SEM (*n* = 6). Data were analyzed by Student’s *t* test (*P < 0.05, **P < 0.01, ***P < 0.001). KC, keratinocytes; Sebo, sebocytes; FAS, fatty acid synthase; HMGCR, HMG-CoA reductase.
(TIF)

S5 Fig Adiponectin was not influence in fully-differentiated sebocytes. (A) A comparison of the level of lipid synthesis using Oil red O staining and the expression of sebocytes differentiation-related gene using quantitative reverse transcription polymerase chain reaction between pre-confluent sebocytes (at 3 days) and post-confluent sebocytes (at 7 days). (B) Detection of intracellular lipids in post-confluent sebocytes treated with adiponectin using microscopy after Oil Red O staining and the change in relative abundance of lipid classes determined by thin-layer chromatography. Scale bars = 20 μm. Data represent means ± SEM (*n* = 6). Data were analyzed by Student’s *t* test (*P < 0.05, **P < 0.01). EMA, epithelial membrane antigen: FADS 2, fatty acid desaturase 2.
(TIF)

## References

[1] Kadowaki T, Yamauchi T, Kubota N, Hara K, Ueki K, Tobe K. Adiponectin and adiponectin receptors in insulin resistance, diabetes, and the metabolic syndrome. J Clin Invest. 2006;116:1784–1792. 10.1172/JCI29126 16823476 PMC1483172

[2] Lago F, Dieguez C, Gómez-Reino J, Gualillo O. Adipokines as emerging mediators of immune response and inflammation. Nat Clin Pract Rheumatol. 2007;3:716–724. 10.1038/ncprheum0674 18037931

[3] Fiaschi T, Magherini F, Gamberi T, Modesti PA, Modesti A. Adiponectin as a tissue regenerating hormone: more than a metabolic function. Cell Mol Life Sci. 2014;71:1917–1925. 10.1007/s00018-013-1537-4 24322911 PMC11113778

[4] Shibata S, Tada Y, Asano Y, Hau CS, Kato T, Saeki H, et al Adiponectin regulates cutaneous wound healing by promoting keratinocyte proliferation and migration via the ERK signaling pathway. J Immunol. 2012;189:3231–341. 10.4049/jimmunol.1101739 22904306

[5] Won CH, Yoo HG, Park KY, Shin SH, Park WS, Park PJ, et al Hair growth-promoting effects of adiponectin in vitro. J Invest Dermatol. 2012;132:2849–2851. 10.1038/jid.2012.217 22739797

[6] Ezure T, Amano S. Adiponectin and leptin up-regulate extracellular matrix production by dermal fibroblasts. Biofactors. 2007;31:229–236. 18997286 10.1002/biof.5520310310

[7] Yamane T, Kobayashi-Hattori K, Oishi Y. Adiponectin promotes hyaluronan synthesis along with increases in hyaluronan synthase 2 transcripts through an AMP-activated protein kinase/peroxisome proliferator-activated receptor-α-dependent pathway in human dermal fibroblasts. Biochem Biophys Res Commun. 2011;415:235–238. 10.1016/j.bbrc.2011.09.151 22024046

[8] Akazawa Y, Sayo T, Sugiyama Y, Sato T, Akimoto N, Ito A, et al Adiponectin resides in mouse skin and upregulates hyaluronan synthesis in dermal fibroblasts. Connect Tissue Res. 2011;52:322–328 10.3109/03008207.2010.528566 21117904

[9] Smith KR, Thiboutot DM. Thematic review series: skin lipids. Sebaceous gland lipids: friend or foe? J Lipid Res. 2008;49:271–281. 10.1194/jlr.R700015-JLR200 17975220

[10] Shi VY, Leo M, Hassoun L, Chahal DS, Maibach HI, Sivamani RK. Role of sebaceous glands in inflammatory dermatoses. J Am Acad Dermatol. 2015;73:856–863. 10.1016/j.jaad.2015.08.015 26386632

[11] Yoshida GJ, Saya H, Zouboulis CC. Three-dimensional culture of sebaceous gland cells revealing the role of prostaglandin E2-induced activation of canonical Wnt signaling. Biochem Biophys Res Commun. 2013;438:640–646 10.1016/j.bbrc.2013.07.129 23948691

[12] Li ZJ, Park SB, Sohn KC, Lee Y, Seo YJ, Kim CD, et al Regulation of lipid production by acetylcholine signalling in human sebaceous glands. J Dermatol Sci. 2013;72:116–122. 10.1016/j.jdermsci.2013.06.009 23849311

[13] Lo Celso C, Berta MA, Braun KM, Frye M, Lyle S, Zouboulis CC, et al Characterization of bipotential epidermal progenitors derived from human sebaceous gland: contrasting roles of c-Myc and beta-catenin. Stem Cells. 2008;26:1241–1252. 10.1634/stemcells.2007-0651 18308950

[14] Tóth BI, Géczy T, Griger Z, Dózsa A, Seltmann H, Kovács L, et al Transient receptor potential vanilloid-1 signaling as a regulator of human sebocyte biology. J Invest Dermatol. 2009;129:329–339. 10.1038/jid.2008.258 18769453

[15] Deepa SS, Dong LQ. APPL1: role in adiponectin signaling and beyond. Am J Physiol Endocrinol Metab. 2009;296:E22–36. 10.1152/ajpendo.90731.2008 18854421 PMC2636986

[16] Ouchi N, Kobayashi H, Kihara S, Kumada M, Sato K, Inoue T, et al Adiponectin stimulates angiogenesis by promoting cross-talk between AMP-activated protein kinase and Akt signaling in endothelial cells. J Biol Chem. 2004;279:1304–1309. 10.1074/jbc.M310389200 14557259 PMC4374490

[17] Smith TM, Gilliland K, Clawson GA, Thiboutot D. IGF-1 induces SREBP-1 expression and lipogenesis in SEB-1 sebocytes via activation of the phosphoinositide 3-kinase/Akt pathway. J Invest Dermatol. 2007;128:1286–1293. 10.1038/sj.jid.5701155 17989724 PMC2902866

[18] Zhou G, Myers R, Li Y, Chen Y, Shen X, Fenyk-Melody J, et al Role of AMPactivated protein kinase in mechanism of metformin action. J Clin Invest 2001;108:1167–1174. 10.1172/JCI13505 11602624 PMC209533

[19] Gerdes S, Rostami-Yazdi M, Mrowietz U. Adipokines and psoriasis. Exp Dermatol. 2011;20:81–87. 10.1111/j.1600-0625.2010.01210.x 21255085

[20] Shibata S, Saeki H, Tada Y, Karakawa M, Komine M, Tamaki K. Serum high molecular weight adiponectin levels are decreased in psoriasis patients. J Dermatol Sci. 2009;55:62–63. 10.1016/j.jdermsci.2009.02.009 19395243

[21] Shibata S, Tada Y, Hau CS, Mitsui A, Kamata M, Asano Y, et al Adiponectin regulates psoriasiform skin inflammation by suppressing IL-17 production from γδ-T cells. Nat Commun. 2015;6:7687 10.1038/ncomms8687 26173479

[22] Nagel G, Koenig W, Rapp K, Wabitsch M, Zoellner I, Weiland SK. Associations of adipokines with asthma, rhinoconjunctivitis, and eczema in German schoolchildren. Pediatr Allergy Immunol. 2009;20:81–88. 10.1111/j.1399-3038.2008.00740.x 18331416

[23] Jeong KY, Lee J, Li C, Han T, Lee SB, Lee H, et al Juvenile obesity aggravates disease severity in a rat model of atopic dermatitis. Allergy Asthma Immunol Res. 2015;7:69–75. 10.4168/aair.2015.7.1.69 25553265 PMC4274472

[24] Salathia NS, Shi J, Zhang J, Glynne RJ. An in vivo screen of secreted proteins identifies adiponectin as a regulator of murine cutaneous wound healing. J Invest Dermatol. 2013;133:812–821. 10.1038/jid.2012.374 23096717 PMC3572451

[25] Kim EJ, Lee DH, Kim YK, Eun HC, Chung JH. Adiponectin Deficiency Contributes to Sensitivity in Human Skin. J Invest Dermatol. 2015;135:2331–2334. 10.1038/jid.2015.150 25880703

[26] Yamane T, Kobayashi-Hattori K, Oishi Y. A high-fat diet reduces ceramide synthesis by decreasing adiponectin levels and decreases lipid content by modulating HMG-CoA reductase and CPT-1 mRNA expression in the skin. Mol Nutr Food Res. 2011;55 Suppl 2:S186–192. 21732532 10.1002/mnfr.201100144

[27] Fu Y, Luo N, Klein RL, Garvey WT. Adiponectin promotes adipocyte differentiation, insulin sensitivity, and lipid accumulation. J Lipid Res. 2005;46:1369–1379. 10.1194/jlr.M400373-JLR200 15834118

[28] Kovács D, Lovászi M, Póliska S, Oláh A, Bíró T, Veres I et al Sebocytes differentially express and secrete adipokines. Exp Dermatol. 2016;25:194–199. 10.1111/exd.12879 26476096

[29] Feingold KR, Elias PM. Role of lipids in the formation and maintenance of the cutaneous permeability barrier. Biochim Biophys Acta. 2014;1841:280–294. 10.1016/j.bbalip.2013.11.007 24262790

[30] Fluhr JW, Mao-Qiang M, Brown BE, Wertz PW, Crumrine D, Sundberg JP, et al Glycerol regulates stratum corneum hydration in sebaceous gland deficient (asebia) mice, J. Invest. Dermatol. 120 (2003) 728–737. 10.1046/j.1523-1747.2003.12134.x 12713573

[31] Choi EH, Man MQ, Wang F, Zhang X, Brown BE, Feingold KR, et al Is endogenous glycerol a determinant of stratum corneum hydration in humans? J Invest Dermatol. 2005;125:288–293. 10.1111/j.0022-202X.2005.23799.x 16098039

[32] Zouboulis CC, Baron JM, Bohm M, Kippenberger S, Kurzen H, Reichrath J, et al Frontiers in sebaceous gland biology and pathology. Exp Dermatol 2008;17:542–551. 10.1111/j.1600-0625.2008.00725.x 18474083

[33] Firooz A, Gorouhi F, Davari P, Atarod M, Hekmat S, Rashighi-Firoozabadi M, et al Comparison of hydration, sebum and pH values in clinically normal skin of patients with atopic dermatitis and healthy controls. Clin Exp Dermatol. 2007;32:321–322. 10.1111/j.1365-2230.2007.02364.x 17335552

[34] Wirth H, Gloor M, Stoika D. Sebaceous glands in uninvolved skin of patients suffering from atopic dermatitis. Arch Dermatol Res. 1981;270:167–169. 7247453 10.1007/BF00408228

[35] Werner B, Brenner FM, Boer A. Histopathologic study of scalp psoriasis: peculiar features including sebaceous gland atrophy. Am J Dermatopathol. 2008;30:93–100. 10.1097/DAD.0b013e31816421fd 18360109

[36] Zouboulis CC, Boschnakow A. Chronological ageing and photoageing of the human sebaceous gland. Clin Exp Dermatol. 2001;26:600–607. 11696064 10.1046/j.1365-2230.2001.00894.x

[37] Pochi PE, Strauss JS, Downing DT. Age-related changes in sebaceous gland activity. J Invest Dermatol 1979;73:108–111. 448169 10.1111/1523-1747.ep12532792

[38] Zouboulis CC. Acne and sebaceous gland function. Clin Dermatol 2005;22:360–366. 10.1016/j.clindermatol.2004.03.004 15556719

