# Correction: Adiponectin Signaling Regulates Lipid Production in Human Sebocytes

**DOI:** 10.1371/journal.pone.0185081

**Published:** 2017-09-14

**Authors:** Yu Ra Jung, Jin-Hyup Lee, Kyung-Cheol Sohn, Young Lee, Young-Joon Seo, Christos C. Zouboulis, Chang-Deok Kim, Jeung-Hoon Lee, Seung-Phil Hong, Seong-Jun Seo, Seong-Jin Kim, Myung Im

Dr. Christos C. Zouboulis should be included in the author byline. He should be listed as the sixth author, and his affiliation is 5: Departments of Dermatology, Venereology, Allergology and Immunology, Dessau Medical Center, Brandenburg Medical School Theodore Fontane, Dessau, Germany. The contributions of this author are as follows: Methodology and Resources.

The correct citation is: Jung YR, Lee J-H, Sohn K-C, Lee Y, Seo Y-J, Zouboulis CC, et al. (2017) Adiponectin Signaling Regulates Lipid Production in Human Sebocytes. PLoS ONE 12(1): e0169824. https://doi.org/10.1371/journal.pone.0169824

The following sentence is missing from the end of the Cell culture section of the Methods: 'Immortalized human SZ95 sebocytes showing morphologic and functional characteristics of normal human sebocytes were maintained and used in the experiments performed as previously described (Zouboulis et al, 1999).

The reference is: Zouboulis CC, Seltmann H, Neitzel H, Orfanos CE. Establishment and characterization of an immortalized human sebaceous gland cell line (SZ95). J Invest Dermatol. 1999;113:1011–1020. doi: 10.1046/j.1523-1747.1999.00771.x PMID: 10594745
