# Supplementary figures and images for: Adiponectin Signaling Regulates Lipid Production in Human Sebocytes
Source: PLoS One. 2017 Jan 12;12(1):e0169824. doi: 10.1371/journal.pone.0169824 (PMC5230785; doi:10.1371/journal.pone.0169824)

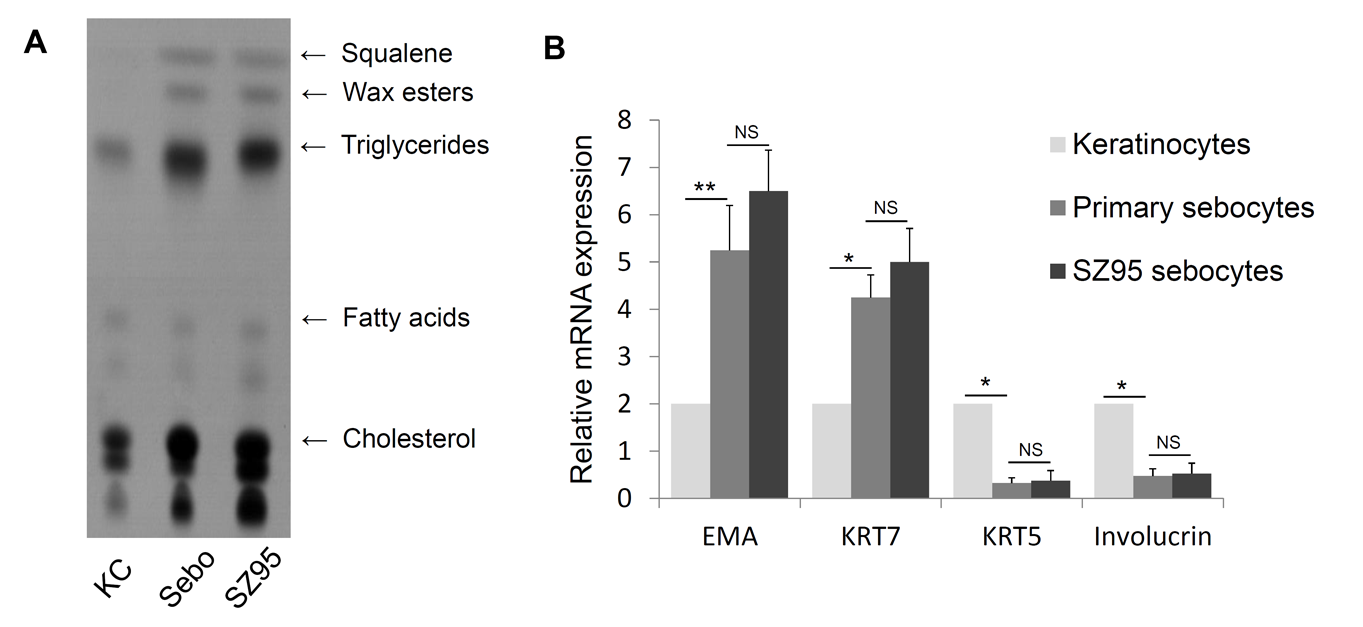

Supplement: S1 Fig — (A) Thin-layer chromatography of neutral lipids extracted from epidermal keratinocytes, primary sebocytes, SZ95 sebocytes, Lipids were identified by comparison with lipid standards. (B) Quantitative reverse transcription polymerase chain reaction analysis of various genes showing sebocytes characteristics. Data represent means ± SEM (n = 7). Data were analyzed by Student’s t test (*P < 0.05, **P < 0.01). KC, keratinocytes; Sebo, primary sebocytes; SZ95, SZ95 sebocytes; EMA, epithelial membrane antigen; KRT, keratin; NS, no significant difference. (TIF) [file pone.0169824.s001.tif]

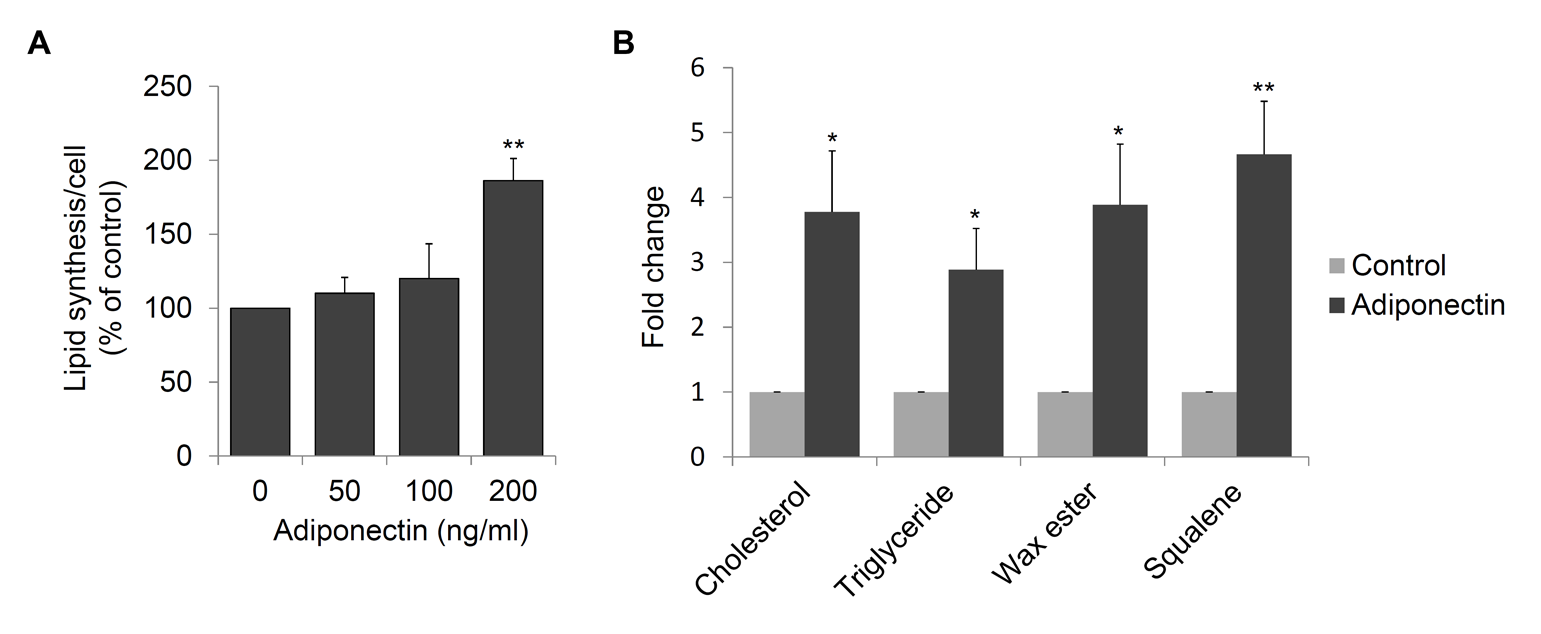

Supplement: S2 Fig — (A) Intracelluar lipid levels in SZ95 sebocytes treated with various doses of adiponectin using Red O staining, calculated as percentages of the value of untreated cells. (B) Relative abundance of lipid determined by thin-layer chromatography. SZ95 sebocytes grown in the presence of [14C]-acetate after treatment with 200ng/ml adiponectin, and changes in specific lipid components were analyzed. Data represent means ± SEM (n = 5). Data were analyzed by Student’s t test (*P < 0.05). (TIF) [file pone.0169824.s002.tif]

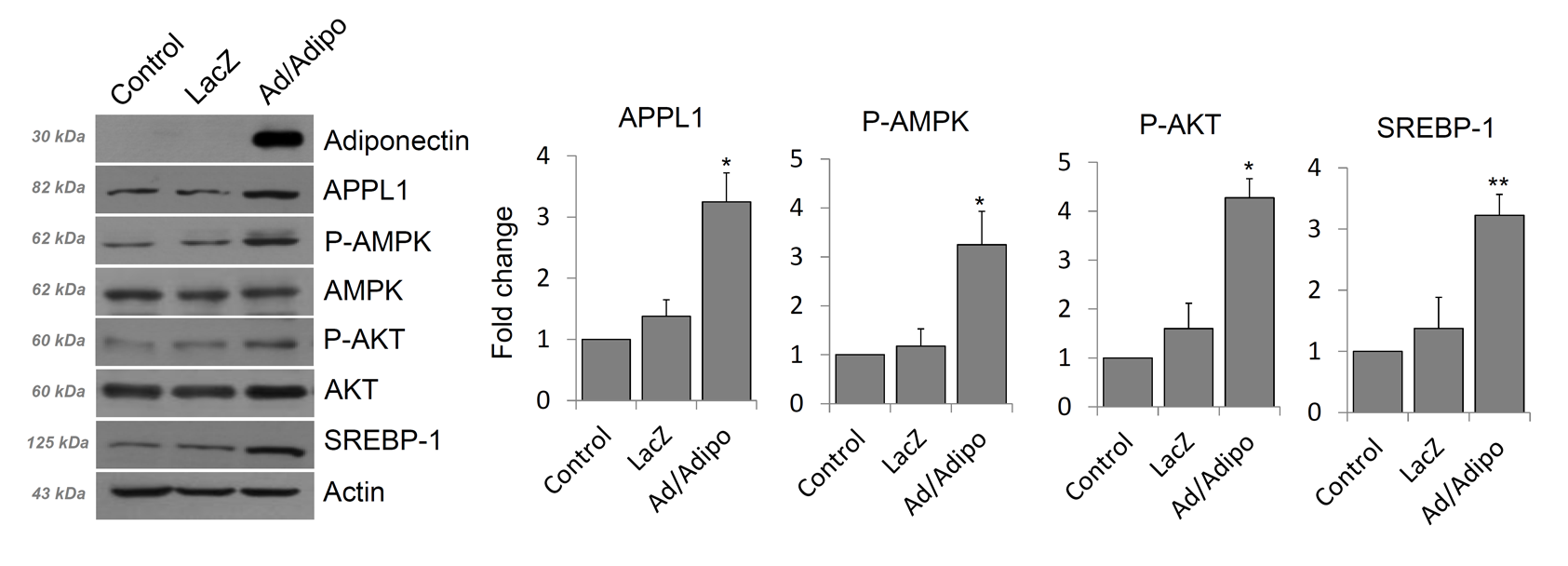

Supplement: S3 Fig — Sebocytes were transduced with an adenovirus expressing adiponectin (Ad/Adipo) or LacZ (control) and expression of related genes was also determined by Western blot analysis. Densitometric analyses of these protein signals were normalized relative to those for actin controls. Data represent means ± SEM (n = 5). Data were analyzed by Student’s t test (*P < 0.05, **P < 0.01). (TIF) [file pone.0169824.s003.tif]

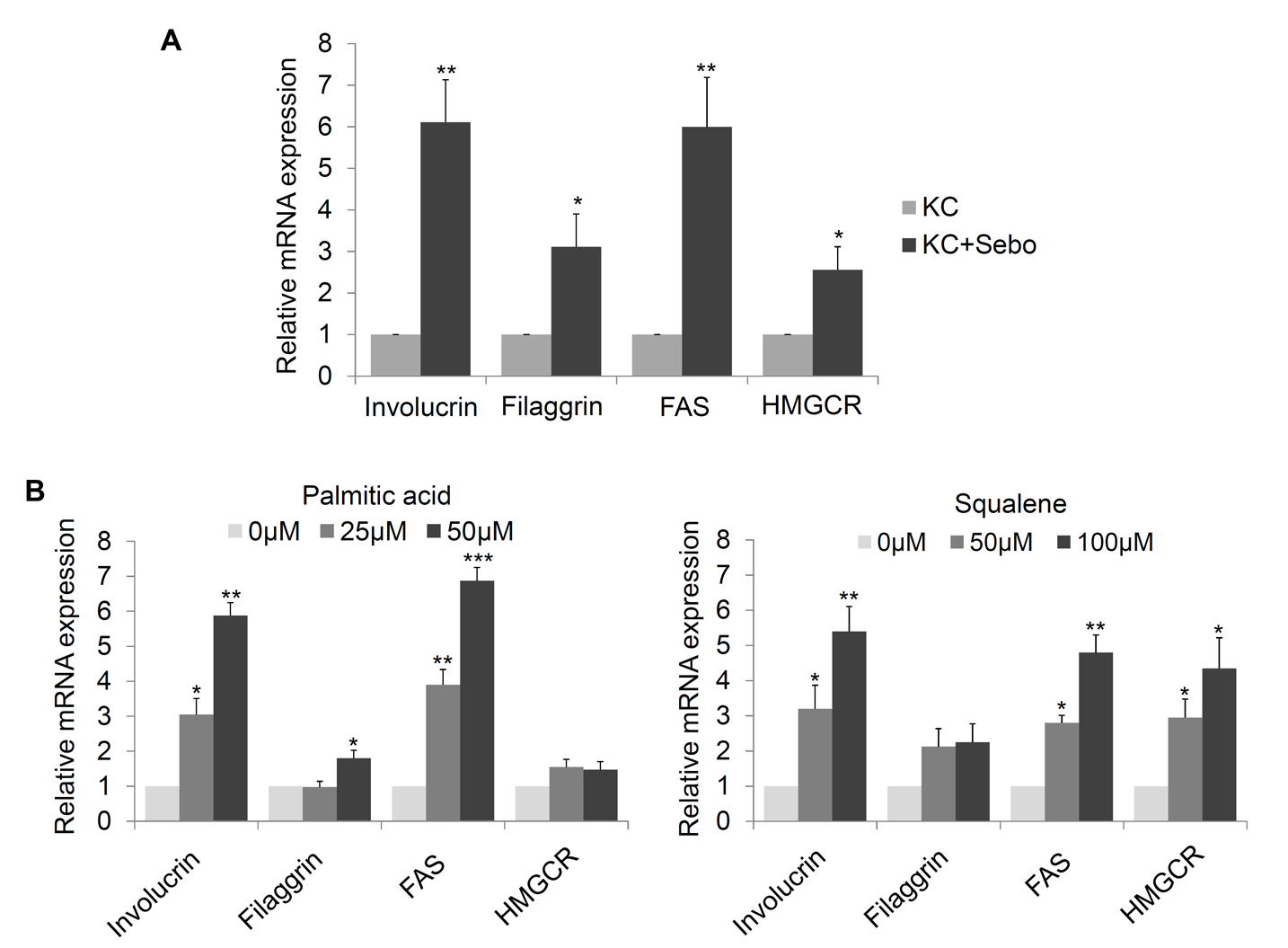

Supplement: S4 Fig — (A) To evaluate the effect of sebocytes lipids on epidermal barrier, keratinocytes from the same donor with sebocytes incubated with and without sebocytes in inserts Quantitative reverse transcription polymerase chain reaction analysis of various genes expressed in keratinocyte and sebocyte co-cultures. (B) Changes in gene expression in keratinocytes after treatment with palmitic acid and squalene, which are major components of sebum in human sebaceous glands. Data represent means ± SEM (n = 6). Data were analyzed by Student’s t test (*P < 0.05, **P < 0.01, ***P < 0.001). KC, keratinocytes; Sebo, sebocytes; FAS, fatty acid synthase; HMGCR, HMG-CoA reductase. (TIF) [file pone.0169824.s004.tif]

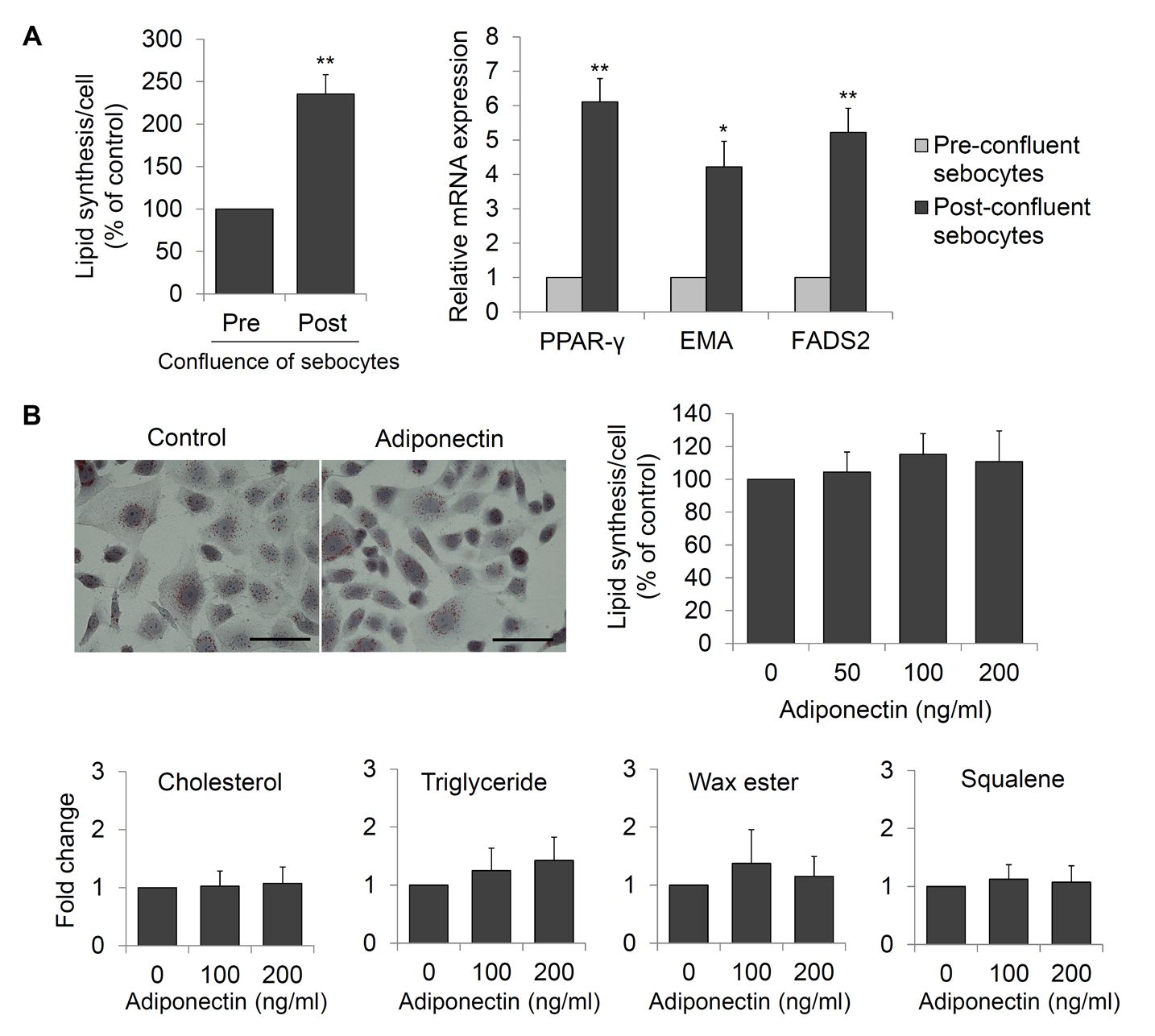

Supplement: S5 Fig — (A) A comparison of the level of lipid synthesis using Oil red O staining and the expression of sebocytes differentiation-related gene using quantitative reverse transcription polymerase chain reaction between pre-confluent sebocytes (at 3 days) and post-confluent sebocytes (at 7 days). (B) Detection of intracellular lipids in post-confluent sebocytes treated with adiponectin using microscopy after Oil Red O staining and the change in relative abundance of lipid classes determined by thin-layer chromatography. Scale bars = 20 μm. Data represent means ± SEM (n = 6). Data were analyzed by Student’s t test (*P < 0.05, **P < 0.01). EMA, epithelial membrane antigen: FADS 2, fatty acid desaturase 2. (TIF) [file pone.0169824.s005.tif]
